# Risk factors of lymph node metastasis in lung squamous cell carcinoma of 3 cm or less in diameter

**DOI:** 10.1097/MD.0000000000007563

**Published:** 2017-07-21

**Authors:** Lijian Huang, Wenshan Li, Lufeng Zhao, Baizhou Li, Ying Chai

**Affiliations:** a Department of Thoracic Surgery; b Pathology Department, 2nd Affiliated Hospital, School of Medicine, Zhejiang University, Hangzhou, China.

**Keywords:** lymph node metastasis, non-small-cell lung cancer, squamous cell carcinoma, systematic lymphadenectomy, tumor marker

## Abstract

Through literature review we cannot find an efficient risk factor of lymph node metastasis in lung squamous cell carcinoma (SCC). This study aimed to investigate the risk factors of pathological lymph node status in patients with lung SCC of 3 cm or less in diameter, to provide some reference for the fellow surgeons in the decision of operative option.

In total, we analyzed 154 patients with lung SCC of 3 cm or less in diameter who underwent lobectomy or bilobectomy or pneumonectomy with systematic lymph node dissection. The relationship between lymph node status and clinical characteristics were examined.

Lymph node metastases were present in 48 patients (31.2%) of the study subjects. Multivariate analysis indicated that, age <60 years old (*P* = .007), tumor location of central-type (*P* = .003), tumor long axis >2 cm but ≤3 cm (*P* = .047) were associated with lymph node metastasis, and their odd ratios (OR) were 3.120, 3.359, and 5.196, respectively. Group analysis of the 74 peripheral lung SCC showed that those with the tumor long axis ≤2 cm had a lower rate of lymph node metastasis (7.9% vs 27.8%, *P* = .025).

Age <60 years old, tumor location of central-type, and tumor long axis >2 cm but ≤3 cm are risk factors of lymph node metastasis in lung SCC. Systematic lymph node dissection or sampling is recommended when tumor central-type location and/or long axis >2 cm in lung SCC are present.

## 1 Introduction

At present, special attention is being paid to lymphadenectomy or lymph node sampling in non-small cell lung cancer (NSCLC) as China's standards for the diagnosis and treatment of primary lung cancer,^[1]^ the US national comprehensive cancer network guidelines,^[2]^ and the European society of thoracic surgeons guidelines.^[3]^ However, current situation is not satisfactory because in many cases lymph node biopsies are not done or done carelessly.^[4–6]^ On the other hand, for the NSCLC that are in a clinical early stage (and most are proved early-stage cancers after the operation), therefore, the question remains: “does indiscriminative complete lymphadenectomy bring unnecessary risks to the patients?” It has been reported that mediastinal lymphadenectomy prolongs hospital stay.^[7]^ In addition, the TNM staging of NSCLC indicates a low invasion potential of certain tumors, in which the probability of mediastinal lymph node metastasis may be even lower.^[8]^ This is why we need to find risk factors of lymph node metastasis for clinical early stage NSCLC.

However, several risk factors of lymph node metastasis were found for lung adenocarcinoma only. The serum level of carcinoma embryonic antigen (CEA),^[9,10]^ the standard uptake value (SUV) in positron emission tomography (PET),^[11–13]^ the tumor size,^[14–17]^ and pathological invasion^[18]^ are the risk factors of lymph node metastasis for clinical stage I lung adenocarcinoma. So far, there are no efficient risk factors of lymph node metastasis in lung squamous cell carcinoma (SCC), including the SUV in PET.^[19,20]^ It is noteworthy that the false positive rate of SUV in predicting lymph node metastasis in lung SCC is considerably high,^[19]^ and that PET is not a routine diagnostic method used in China.

This study aims to analyze the factors that are associated with lymph node metastasis of lung SCC of 3 cm or less in diameter. Our study aims to provide some reference for the decision of removal of lymph nodes in a patient with or without risk factors.

## 2 Methods

This study was conducted in accordance with the Declaration of Helsinki and relevant policies in China. The study protocol was approved by the local Institutional Review Board and the Ethics Committee of the Second Hospital Affiliated to the Medical School of Zhejiang University. Further patient consent was waived because the data were analyzed retrospectively.

### 2.1 Patients

In this study, 2358 consecutive lung cancer patients were screened who were admitted to the Department of Thoracic Surgery at the Second Hospital Affiliated to the Medical School of Zhejiang University from January 2010 to December 2015. Inclusion criteria used are: lung SCC confirmed by pathology; the long axis of tumor ≤3 cm; patients who received lobectomy, combined lobectomy, or pneumonectomy; patients who underwent intrapulmonary, hilar, and mediastinal lymphadenectomy. A total of 176 patients with lung SCC met the above criteria.

Exclusion criteria: coexisting hydrothorax or distal metastasis (n = 5); the short axis of lymph node >1 cm on computed tomography (CT) (n = 5); a history of preoperative chemotherapy or radiotherapy (n = 8); a history of malignant tumor (n = 4). After exclusion, a total of 154 patients with clinical stage I (cT1-2N0M0) lung SCC were selected and included in the study.

### 2.2 Preoperative investigations

Before the operation, all the patients underwent a contrast-enhanced chest CT with high resolution. In addition, electrocardiogram, pulmonary function examination, brain magnetic resonance imaging or CT, whole-body bone scan, ultrasound imaging of the abdomen, adrenal gland and superficial lymph nodes, and detection of serum tumor markers were all conducted. Due to the high costs, a PET check was only performed in a minority of the patients (data not shown).

### 2.3 Surgical procedures

All patients underwent at least lobectomy plus intrapulmonary, hilar, and mediastinal lymphadenectomy. In addition, combined lobectomy (2 cases), sleeve lobectomy (1 case), or pneumonectomy (2 cases) followed by systematic lymphadenectomy was performed in a few cases due to the central location of the tumors. The extent of systematic lymphadenectomy included dissection of lymph node stations 4 to 14 for left lung cancers, and dissection of lymph node stations 2 to 4, and 7 to 14 for right lung cancers.

Lymphadenectomy was conducted based on the anatomical structure, and the entire lymph nodes and the surrounding adipose tissues were removed to reach skeletonization. If the lymphadenectomy was conducted difficultly via a thoracoscope, then the surgeons turned into thoracotomy to ensure the thoroughness of lymph node dissection. The intrapulmonary dissection of lymph nodes done by the surgeons is common after the lung resection.

### 2.4 Evaluation of pathologic findings

Pathological assessment of the hematoxylin and eosin (H&E)-stained specimens was done by a same pathologist (BL) and a random pathologist in a blinded manner. Pathologic node stage was classified as pN0, pN1, or pN2. And pN0 represents no lymph node metastasis, pN1 represents N1 (Stasions 10–14) lymph node metastasis, and pN2 represents N2 (Stasions 2–9) lymph node metastasis, respectively.

### 2.5 Statistical analyses

Clinical data of 154 patients were recorded and included age, sex, smoking history, length of the long axis of the tumor, tumor location (the lung SCC was considered to be peripherally located if tumor was within 20 mm of the costal pleura, otherwise it was considered to be centrally located),^[21]^ levels of serum tumor markers such as CEA (normal <5 ng/mL; abnormal ≥5 ng/mL), squamous cell carcinoma antigen (SCC-Ag) (normal <1.5 ng/mL; abnormal ≥1.5 ng/mL) and cytokeratin fragment 21-1 (CYFRA21-1) (normal <5 ng/mL; abnormal ≥5 ng/mL), and lung SCC histologic subtype which could be obtained by preoperative biopsy.

The above clinical factors and postoperative lymph node metastasis were analyzed by univariate analysis, *χ*^2^ test, or Fisher exact test. When a significant difference was found between multiple groups, the *P* value corrected by the Bonferroni method was used to compare between 2 groups. Multivariate logistic regression analysis was performed on the factors that showed significance in the univariate analysis. The potential predictive factors described as quantitative data were analyzed by the receiver operating characteristic (ROC) curve. All the analyses were conducted using SPSS 22 statistic software (IBM Corp, Armonk, NY). *P* < .05 was considered statistically significant.

## 3 Results

### 3.1 General clinical characteristics

The tumors were classified as subsolid in 1 patient (0.6%) and solid in 153 (99.4%). The average age of 154 patients (144 males and 10 females) was 63.4 years (ranging from 42 to 82 years). A total of 4556 lymph nodes were dissected from 154 enrolled patients, and the average number of N1 and N2 lymph nodes dissected from each patient was 29.6. Lymph node metastasis occurred in 48 patients (31.2%), including 28 cases of pN1 (5 cases with intrapulmonary lymph node metastasis only, involving lymph node stations 13 and/or 14) and 20 cases of pN2. The number of lymph nodes containing metastatic tumor cells was 152, which accounted 3.3% of total lymph nodes dissected. Among the metastatic lymph nodes, there were 112 pN1 lymph nodes, which accounted 73.7% of all metastatic lymph nodes.

### 3.2 Univariate analysis

Univariate analysis (Table 1) showed that age, length of the long axis of tumor, tumor location, SCC-Ag level, CYFRA21-1 level were significantly associated with lymph node metastasis in lung SCC. There was a significant difference between the lung SCC with the long axis of tumor >2 cm and those ≤2 cm.

**Table 1:**
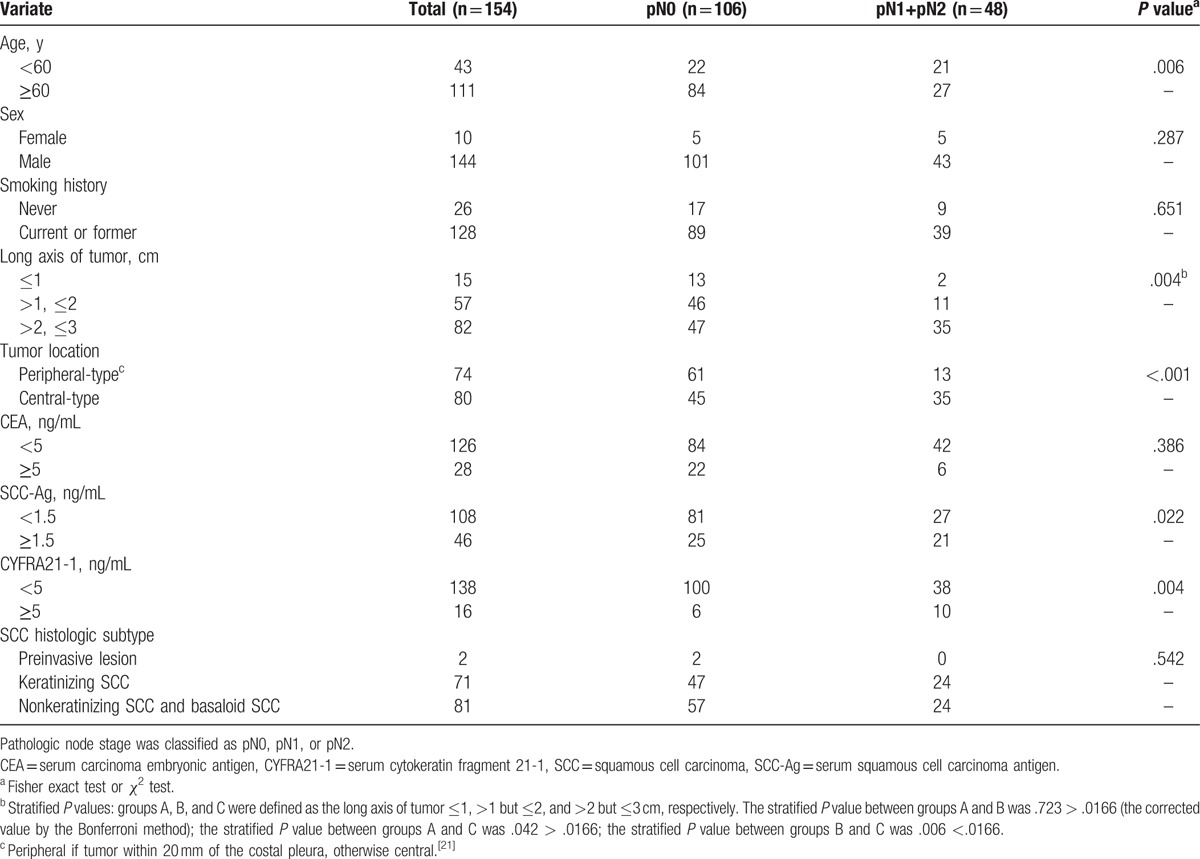
Univariate analysis showing the association between the clinical characteristics of 154 lung squamous cell carcinoma of 3 cm or less in diameter and lymph node metastasis.

The data indicated that the central SCC had a higher probability of lymph node metastasis compared with the peripheral SCC. Univariate analysis of the 74 peripheral SCC (Table 2) showed a significant difference between those with the tumor long axis >2 cm and those with the tumor long axis ≤2 cm (*P* = .025). In only 3 patients with peripheral lung SCC with tumor long axis ≤2 cm (3/38, 7.9%) lymph node metastasis could be detected.

**Table 2:**
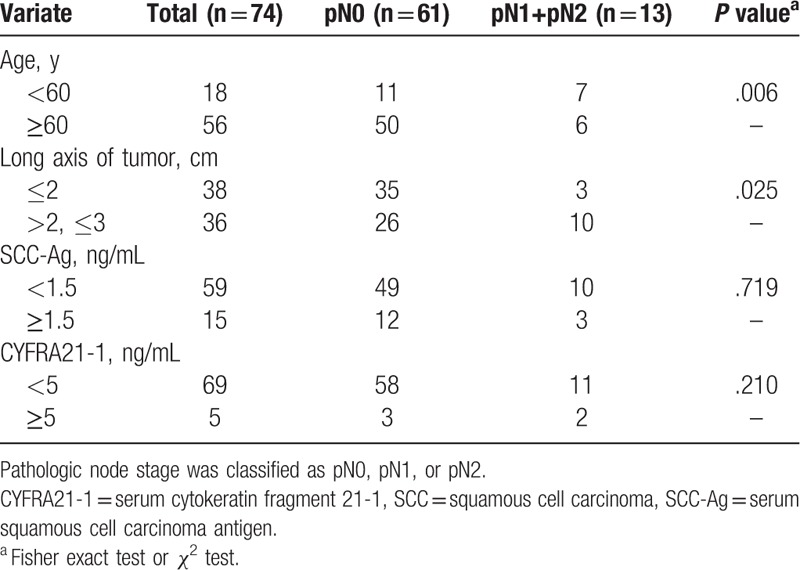
Univariate analysis showing the association between several clinical characteristics of 74 peripheral lung squamous cell carcinoma of 3 cm or less in diameter and lymph node metastasis.

In the stratified analysis of pN1 and pN2 lung SCC (Table 3), tumor long axis >2 cm and central location significantly increased the rate of N1 metastasis (all *P* values were = .001). On the other hand, age < 60 years significantly increased the rate of N2 metastasis (*P* = .004).

**Table 3:**
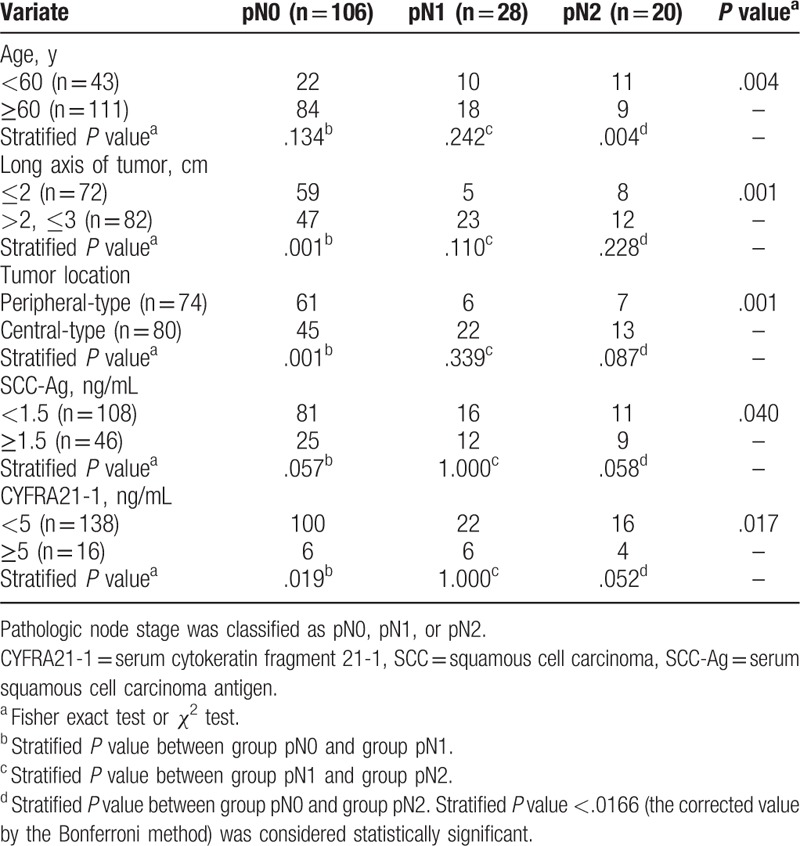
Stratified analysis of N1 and N2 lymph node metastasis in 154 lung squamous cell carcinoma of 3 cm or less in diameter.

**Figure 1:**
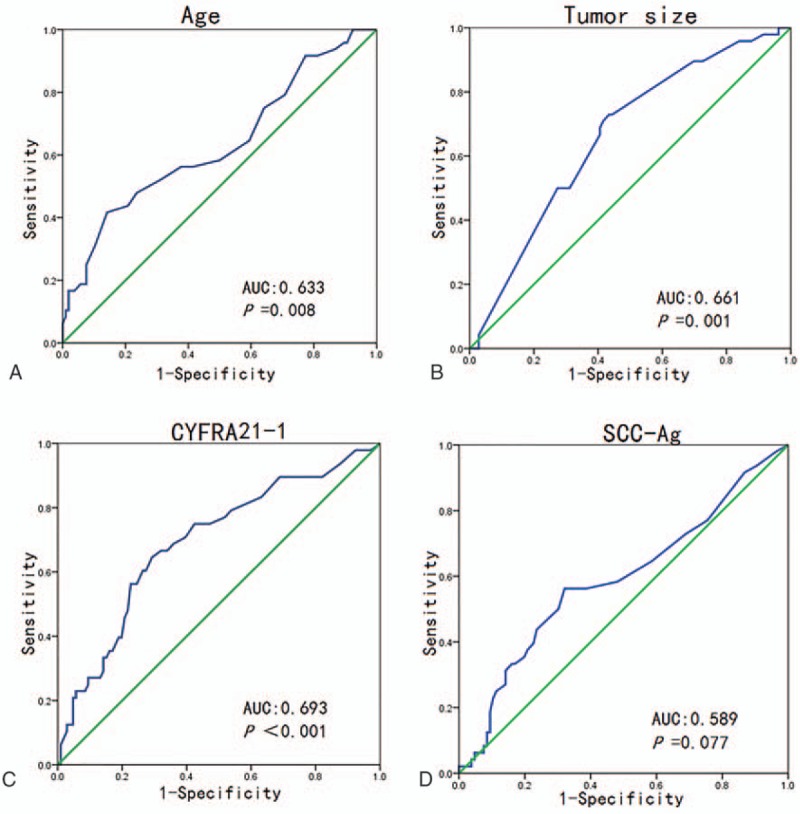
Area under the receiver operating characteristic curve values (AUC) used for predicting lymph node metastasis in lung SCC. A, Age: AUC, 0.633 (95% CI, 0.534–0.732), *P* = .008. B, Tumor size: AUC, 0.661 (95% CI, 0.572–0.751), *P* = .001. C, CYFRA21-1: AUC, 0.693 (95% CI, 0.601–0.784), *P* < .001. D, SCC-Ag: AUC, 0.589 (95% CI, 0.488–0.690), *P* = .077. SCC = squamous cell carcinoma, SCC-Ag = squamous cell carcinoma antigen, CI = confidence interval, CYFRA21-1 = serum cytokeratin fragment 21-1.

As potential predictive factors of lymph node metastasis, age, length of tumor long axis, levels of SCC-Ag, and CYFRA21-1 were analyzed by using ROC curves (Fig. 1). The data showed that age (*P* = .008), length of tumor long axis (*P* = .001), and CYFRA21-1 level (*P* < .001) could be predictive factors of lymph node metastasis in lung SCC. The area under the ROC curve (AUC) of these 3 factors was 0.663 (95% confidence interval (CI) = 0.534–0.732), 0.661 (95% CI = 0.572–0.751), and 0.693 (95% CI = 0.601–0.784), respectively.

### 3.3 Multivariate analysis

Five factors including age, length of tumor long axis, tumor location, SCC-Ag level, and CYFRA21-1 level were subjected to multivariate analysis of the 154 patients. The results indicated that age <60 years old (*P* = .007), tumor location of central-type (*P* = .003), tumor long axis >2 cm but ≤3 cm (*P* = .047) were associated with lymph node metastasis, and their odd ratios (OR) were 3.120, 3.359, and 5.196 respectively (Table 4).

**Table 4:**
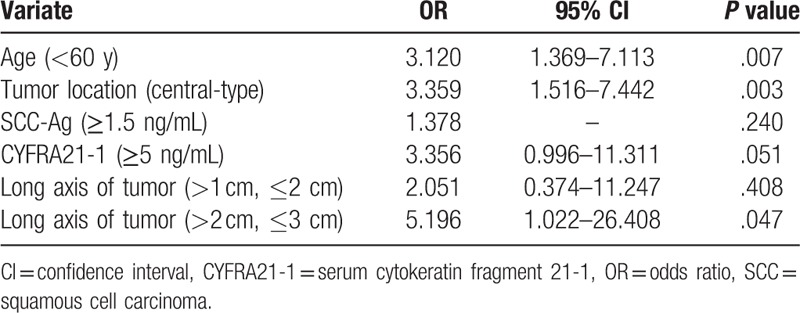
Multivariate analysis for related and predictive factors of nodal involvement in 154 lung squamous cell carcinoma of 3 cm or less in diameter.

## 4 Discussion

At present, the incidence of lung cancer is high. Along with the popularization of CT, early stage lung cancers without clinical symptoms are diagnosed in a rising trend.^[22]^ Among the subtypes of NSCLC, adenocarcinoma has the highest incidence rate, and SCC accounts for about 20% to 30% of NSCLC.^[20]^ Unfortunately, few advances have been made on nodal involvement research in lung SCC.

Through literature review, we identified only 1 report about the risk factors of lymph node metastasis in lung SCC. Among tumor size, SUV in PET, serum levels of CEA, and CYFRA21-1, no factor showed a significant risk value in Tsutani et al's study.^[20]^ We believe that the thoroughness of lymphadenectomy (including intrapulmonary, hilar, and mediastinal lymphadenectomy) is critical in these types of studies. If the false negative rate of lymph node metastasis turns out to be high, the results will be unreliable. In our institution, lobectomy followed by systematic lymphadenectomy has strictly been practiced for confirmed lung SCC since January 2010. Thus, lobectomy was set as one of the inclusion criteria and did not cause bias for selection. It has been reported that the anatomic number of bilateral mediastinal lymph nodes is 23.^[23]^ In our study, the average number of intrapulmonary, hilar, and mediastinal lymph nodes in the enrolled patients was 29.6, including lymph node stations 4 to 14 in the left lung cancers, and lymph node stations 2 to 4 and 7 to 14 in right lung cancers. Lymphadenectomy is therefore considered acceptable. In our study, the rate of lung SCC lymph node metastasis (48/154, 31.2%) is higher than Tsutani et al^[20]^ previously reported (12/100, 12%), and this may be the reason for the 2 different results.

It is noteworthy that the different rates of lung SCC lymph node metastasis in 2 studies could also be related to the difference of preoperative mediastinal or hilar lymph nodes staging. In our study, preoperative lymph node positivity was defined as at least 1 lymph node with a short axis measuring more than 1 cm on CT, that is same with Ye et al's study,^[24]^ but in Tsutani et al's study^[20]^ preoperative lymph node positivity was defined as a presence of >1 cm enlargement in mediastinal or hilar lymph nodes on CT or a presence of >1.5 accumulation for maximum standardized uptake values (SUVmax) in these lymph nodes on PET/CT, respectively. Seventy-six patients (76%) received PET/CT in their study, but only 6 patients (3.9%) received PET/CT in our study, whose SUVmax of lymph nodes were also <1.5.

We have found the relationship between lymph node status and the tumor invasiveness such as pleural invasion, vascular invasion, and perineural invasion (*P* values were .001,.003,.001, respectively). But the diagnosis of the tumor invasiveness is difficult to determine before or during surgery. So they are not listed in our study. As stated earlier, we found 3 preoperative risk factors in multivariate analysis in the current study. Lymph node dissection or sampling is encouraged when these risk factors of lymph node metastasis are present. Intriguingly, when the patient's age was below 60 years, N2 metastasis was more likely to happen. By contrast, N1 metastases were more likely to occur when the tumor long axis was above 2 cm or when the tumor was in a central location. The influence of age on lymph node metastasis may need to be further evaluated with a larger number of cases. In close proximity to hilar and intrapulmonary lymph nodes, central-type tumors were prone to N1 metastasis via the deep lymphatic plexus next to the bronchus.^[25]^ The rate of N1 metastasis increased significantly when the tumor was above 2 cm, suggesting that sublobar resection is not sufficient for these cases due to the omission of hilar and intrapulmonary lymphadenectomy.

Group analysis of the 74 peripheral lung SCC indicated that the age (*P* = .006) and the length of tumor long axis (*P* = .025) were significant factors. Our results suggest that the peripheral lung SCC with long axis is ≤2 cm may be a precondition for sublobar resection with lymph node dissection or sampling. This conclusion is partly consistent with the study of Flores et al,^[21]^ which showed that peripherally located NSCLCs manifesting as a solid nodule that are less than 20 mm in diameter should be considered candidates for sublobar resection without mediastinal lymph node resection. However, even in the group of the 38 peripheral lung SCC with tumor long axis ≤2 cm, there were 3 exceptions with nodal involvement (7.9%, pN1 in 1 patient and pN2 in 2), and the long axis of the smallest one was only 0.7 cm. Lymph node dissection or sampling is still recommended in these cases independently of resected lung volume (lobectomy or sublobar resection).

In the study of clinical stage IA lung SCC by Tsutani et al,^[20]^ the results indicated no significant association of CEA or CYFRA21-1 with lymph node metastasis. Moreover, Cho et al^[26]^ found that CYFRA21-1 was associated with N1 metastasis, but not with N2 metastasis in NSCLC, whereas SCC-Ag was not found to be associated with either N1 or N2 metastasis. In multivariate analysis of the present study, we could not find a predictive value of CYFRA21-1 for lymph node metastasis in lung SCC (*P* = .051, slightly greater than.05), and the stratified analysis indicated that it was not significant in N1 metastasis (*P* = .019, slightly greater than.0166). We hypothesize that an increased sample size may lead to new findings.

Age, length of tumor long axis, and levels of SCC-Ag and CYFRA21-1 were analyzed as potential predictive factors using ROC curves. The results indicated that age, length of tumor long axis, as well as CYFRA21-1 levels could be used as preoperative predicting factors of lymph node metastasis in lung SCC; however, the prediction power of each single factor was not strong. These findings suggest combined prediction of multiple factors. ROC curve analysis of the value of the length of tumor long axis multiplied by the absolute value of CYFRA21-1 level gave an AUC value of 0.723 (95% CI = 0.638–0.807; *P* < .001), suggesting that the combined use of multiple predictive factors can increase the prediction power.

Our findings may provide some reference for the fellow surgeons in the decision of operative option. However, there are some defects in our study that need to be addressed. First, this study was retrospective, but not prospective, and single-centered. Second, the sample size was not very big, and there were only 48 cases of lymph node metastasis included in the study. This may have affected the outcome of logistic regression analysis (any bias in the OR = 3.120 of age < 60 vs ≥ 60 years?). Moreover, because of the limited sample size, the samples were even more diluted in the stratified analysis, which may cause bias. Third, this study only focused on lung SCC, and our conclusion is not applicable to lung adenocarcinoma, which has a higher incidence rate. Sometimes, lung SCC is not diagnosed until surgery and only confirmed during or after the surgery. In addition, few patients are willing to take the PET/CT. The associated costs are very high and not covered by medical insurance in China. Although it has been reported PET/CT provides few reference values for lung SCC,^[19,20]^ our study lacked the PET/CT data for validation and control.

In conclusion, among the patients with lung SCC of 3 cm or less in diameter, age <60 years, tumor location of central-type, tumor long axis >2 cm but ≤3 cm all significantly increased the rate of lymph node metastasis. In addition, the level of CYFRA21-1 might also be used as a predictive factor of lymph node metastasis in lung SCC. The influence of age on lymph node metastasis may need to be further evaluated. Systematic lymph node dissection or sampling is recommended when tumor central-type location and/or long axis >2 cm in lung SCC are present. Lymph node dissection or sampling is still recommended in peripheral lung SCC with tumor long axis ≤2 cm, yet this needs to be further verified in prospective studies with larger study groups.
